# Maxims of Military Surgery

**Published:** 1861-11

**Authors:** 


ART. II.—*Maxims of Military Surgery.* (*Maximen der Kriegsheilkunst.*) By DR. L. STROMEYER, etc. Hanover, 2 vols., pp. 773, 1858; and Supplements, pp. 150, 1861.

[Continued from the May number, p. 425.]

*Wounds of the head.*—In the campaigns of Dr. Stromeyer, these were nearly all from projectiles: the country being so much intersected by small streams as to render cavalry of very little effect; and, in consequence, few sabre wounds presented themselves. Injuries of the head from falls, or from blows with the butt-end of the guns, were not numerous. It is natural, says our author, that one long accustomed to injuries of the head from civil practice should suppose that those received in war would exhibit the same phenomena, but in an intenser degree; and to be surprised to find that this is by no means the case. The essential difference consists in the comparative insignificance, with gunshot wounds, of the symptoms of concussion, and, what seems probably dependent on this, in the more local form, of the symptoms of reaction. It may be difficult to present this in a statistical form, as the slightest injuries received in civil life generally pass at once under medical attention, while wounds received in battle are immediately carried to the hospital. The slow, insidious inflammation which not unfrequently follows injuries of the head received in ordinary practice, has only been met with by Dr. Stromeyer, among several hundred injuries of slighter severity received from bullets, in a single instance. It cannot be urged, in reply to this, that early reception into hospitals protects men against these unfavorable incidents; for very many of the slightly wounded do not report themselves as ailing, or leave the hospital in a few days, from which practice Dr. Stromeyer has not met with a single example of any evil result. It is only necessary to read Pott's cases to be convinced that a short residence in a hospital affords inadequate protection against the evil consequences of a concussion from a ball, without wound or fracture. The severe phenomena of concussion, which arise from violence committed with blunt bodies moved with less velocity than bullets, are wanting in gunshot wounds. The injury from these is inflicted with so much rapidity as not to affect a wide surface, or produce an "actio in distans;" or, at least, so as to achieve these consequences in a very slight degree.

The patient wounded with a bullet in the head sinks to the ground, but soon recovers himself. If he afterward becomes unconscious, it is not the symptoms of concussion that are agents in the case, as frequently happens with those struck by a club. If these last are bled too soon or too freely, the original symptoms of concussion recur. It is from the small importance of its symptoms, however, in shot wounds of the head, that intellectual and critical observers, such as John Hennen, have been induced to devote so little attention to the concussion, and hold the discrimination of it to be of no practical value. Civil practitioners, and those who, like Guthrie, have, at the time of writing, enjoyed large civil experience subsequently to military, judge very differently. The danger from shot wounds of the head is not in proportion to the remote sympathies awakened, but to the amount of injury undergone by the party struck by the weapon. Every soldier who had a portion of the skull, exceeding three or four square inches, crushed, in the experience of Dr. Stromeyer, perished on the field of battle.

Extensive shattering of the skull, with laceration of the brain, produces death so speedily, that little opportunity is afforded for investigating the phenomena; the more so as the attention of the surgeon, in the first days after a battle, is so intensely demanded by other cases. The bleeding is often important, especially where the sufferer is carried to a distance, masses of the brain protrude, and the subject sometimes revives from the apparent approach of death, to continue for a day or two in a state of unconsciousness, not always accompanied with palsy of particular limbs.

Gunshot wounds of the head, on account of their frequency, and of the benefit to be derived from an appropriate treatment, are well worthy of the greatest care and attention.

Conical and spherical bullets inflict the same injuries; but the discrimination between them can frequently be made from the shape of the wound in the bone, or of the portion of pericranium which is stripped off.

*Case where the ball goes quite through the head.*—Dr. Stromeyer has never seen life preserved, in a single instance, where a bullet had passed through any of the larger diameters of the skull. Of wounds penetrating through smaller segments, he has seen recoveries. The shorter the passage through the brain, and the more remote the route from the base of that organ, the longer may life continue; and this may extend to weeks, or even months. Yet he has never seen a recovery, where the wound or the parts adjacent formed an abscess. The ball drives the fragments of bone into the brain; and sinuses generally form on various sides of both the point of entrance and that of exit. Yet, in these frightful wounds, the activity of the heart is only depressed for a short interval; the pulse becomes full and hard, effusions of blood become mixed with the crushed brain, and blood and brain not unfrequently issue together from the wound, when the latter is sufficiently gaping.

Men who had received wounds of the first-mentioned, central description, not unfrequently, where the distance was long, died in the transportation wagons. "Without doubt," he observes, "the jolting of a wagon is the best means of dismissing such unfortunate individuals from the world;" "and certainly that which contributes so much to accelerate the termination of fatal cases, must be well calculated to aggravate injuries of a more moderate character."

"On the 6th of April, the Hanoverian soldier, B., received a ball in the left temple, which pierced the os frontis a little above the suture with the parietal and the greater wing of the sphenoid, crushed the cribriform plate and right smaller wing of the latter, and escaped through the right orbit. At the point of entrance of the missile, brain escaped, and from that of exit the lachrymal gland hung protruded. The patient was transported four miles. Symptoms of concussion were not present; but the sufferer was fatuitous, blind, and deaf. He could move all his limbs. He roared or screamed continuously, day and night, for two months. It sometimes seemed as if he observed the presence of light. He ate, with great eagerness, whatever was placed in his mouth. He ceased to scream a short time before his death, which took place on the 5th of June. The whole route of the ball was converted into an abscess."

*The ball penetrates into the brain.*—It is not always the case that a ball is imbedded in the interior of the head, where first external appearances give that impression. A gaping wound may exist in the skull, and the finger or probe may pass without impediment to a considerable depth into the brain. The ball has shattered the part of the skull on which it impinged, driven the fragments deep into the organ, and then fallen to the ground on the outside, from its motive force being exhausted before it sunk beyond its middle. This case has occurred to Dr. S. several times; and the head has been opened and no bullet found. If a cure take place under such circumstances, of which the writings of military surgeons furnish examples, it has been believed that the bullet has been healed over. In the opinion of the author, those cases only should be reckoned as having undergone this, in which the patient has subsequently died, and a bullet been found.

The phenomena produced by this lesion vary according as the ball has effected much destruction in its ingress, or has been easily stopped and remained arrested in the brain. In the following cases, the symptoms may appear inconsiderable:—

"On the 10th of September, 1850, a Danish soldier was brought to Rendsburg, in whom a bullet had entered through the parietal bone near the lambdoidal suture. The wound in the cranium admitted the fore-finger without difficulty, and all the pieces were driven into the brain. Neither vomiting nor any of the other indications of a concussion of any continuance had been present. The patient saw and heard, and could move all his limbs, but was dumb. When left quite without interference, he lay quiet, but did not sleep much. Under an antiphlogistic treatment, this state of things continued ten days. A large part of the splinters were brought by the motions of the brain to so close an approximation with the interior of the cranium that they could be easily removed with the forceps, and without the application of any force. After this little operation, however, the patient's restlessness greatly increased; and he died on the next day. The ball had been driven into the left anterior lobe of the brain. The cerebral substance over the track of the ball was decomposed and putrid, and beyond this was inflammatory softening. The brain was hyperæmic throughout."

"The much-lamented Captain von D. received, on the 23d of May, 1849, a rifle-bullet, which passed through the leather escutcheon of his cap, and penetrated his left temple. Insensible as he was, he was placed in a wagon to be removed to a house at a distance, rendering necessary a journey of half an hour. The wagon had scarcely started when he became affected with clonic spasms of all his limbs. As soon as he was quieted and becomingly disposed, the same attempt was repeated, with the same result. He was then placed on a handbarrow, and carried to the place proposed, without any repetition of the spasms. I saw him in the afternoon. The ball had entered the parietal bone one and a quarter inches above the zygomatic process. On the opposite temple, a little nearer the zygomatic process, was felt, under a somewhat pasty swelling, a crepitating fragment of bone, of considerable size. The eyelids were swelled to bursting with extravasated blood; and this phenomenon took place immediately after the reception of the wound; thus authorizing us to conclude that the orbit was shattered. Vomiting and other evidences of concussion were not present. There was no palsy of the limbs; hearing was entire; and consciousness in no degree destroyed. The wounded man did everything we wished. When told that he was to be bled, he pulled off his coat, and, at the close, assisted in drawing it on again. A venesection of eight ounces was undergone without any observable sinking of the pulse. At a later period, he frequently took up the vessel to receive his urine; and he held the glass when he drank. Sometimes, however, his urine escaped involuntarily. The most remarkable occurrence, in these respects, was that he had lost the control of his organs of speech. Notwithstanding abundant evidence of the continuance of consciousness, he gave not the slightest answer to the questions that were put to him; although, at the same time, he showed unmistakably enough that he understood them. On the other hand, he was heard to utter, from time to time, the involuntary cry of pain, 'Oh, God! yes.' Thus the connection between his organs of thought and the nerves of those of speech was interrupted; and there ensued, in the latter, involuntary efforts at action by the expression of pain which has become so common in the north of Germany.

"In this case, it would not have been impossible to extract the ball, not by the narrow wound of entrance, but by the opposite side, where it had loosened so large a piece of bone. It would have only been found necessary to lay bare and remove this, to obtain access to the ball; which, possibly, might have laid close behind it. Yet I did not think this advisable. It should be considered a prostitution of surgery, when operations are performed in such hopeless cases. The anterior lobes of the brain were evidently crushed, and the upper parietes of the orbits shattered. The extraction of the ball could only accelerate death, promoting, as it would, the decomposition of the contused brain by the access of air.

"Death ensued on the 20th of May. On dissection, I found that, from the wound of entrance in the skull, three fissures radiated in different directions; and that a small portion of the ball had been scraped off by the bone. Near an inch and a half of each of the anterior lobes was crushed by the ball and the fragments of bone, and was converted into a paste with half-coagulated blood. The ball, an irregularly disfigured rifle-bullet, lay upon the cribriform plate. Probably it was the motion of this, during carriage by the wagon, that occasioned the spasms. The width of the fragment, from the inside of the parietal bone and of the ala magna sphenoidei, was one and a half inches; so that the extraction of the ball by this route had been possible. But, from its altered shape, the task of certainly and safely finding the latter would not have been so easy. We made the attempt on the corpse, prior to the section of the skull; but gave it up, from wishing to avoid moving the parts from their original position. The extraction of the ball, in these circumstances, could, in no case, answer any useful purpose, as there were innumerable small splinters of bone buried in the brain, which could not have been removed at the same time."

"The greatest amount of destruction in the brain, produced without the immediate cessation of life, and with a partial preservation of consciousness, was that of a man shot in the forehead as he was going down stairs. He insisted tenaciously that he had fallen down stairs, and thrust a splinter into his forehead. Portions of brain were forced from the wound, and *a probe was introduced six inches.* His pulse and temperature were natural; he moved his limbs automatically; and he frequently grasped at the injured part. He often answered questions quite correctly, but sometimes not at all; and during long ones, seemed to fall asleep. No one could induce him to tell his age. He became gradually more sleepy; and died in twelve days. The ball had passed over the superior arch of the orbit, and lodged within the dura mater, above the transverse ridge of the os occipitis. Its route was filled with splinters, crushed brain, blood, and pus; and among the splinters was one of the smaller wings of the sphenoid bone. Two fissures extended below the base of the brain."

Cases of this kind have occurred to Dr. Stromeyer a number of times; but he has seen none in which life was preserved. "To these are, in general, most nearly analogous some thousands of cases met with in great wars, among which we at once stumble upon a case that unites all favorable circumstances; perhaps attended by physicians who are not afraid to bleed, as our young German Æsculapiuses now are." Yet Thompson found no single recovery of such among all the wounded of Waterloo.

*The ball remains stuck in the wound of the skull.*—The difference between this injury and the one last enumerated is much less than might be supposed. It is true, the ball can be removed; but this is of by far the less advantage, as the fragments of the penetrated bone are generally forced through the dura mater and driven into the brain. This is more certain to happen if the ball strikes at a right angle. A shot impinging at an oblique angle may depress the inner table without seriously injuring the dura mater. In injuries of this class, it is not uncommon for the sharp edge of the broken bone to split the ball quite through.

Principal Stromeyer gives two very curious and instructive cases under this section of his subject, which our space will not suffer us to copy without abridgment.

In one of these, the ball actually fell out and was lost, between the inspection of the surgeon of the battalion on the field of conflict and that of his chief at the hospital; and the stellated form and the appearance of the wound rendered it hard to believe, when Dr. Stromeyer saw it, that such a depression had really contained a ball. The elasticity of the bone and the distending parts, and the pulsations of the brain, will suggest themselves to the reader's mind; but Dr. Stromeyer hazards no hypothesis as to the cause of this change. The patient possessed full consciousness and the use of his limbs. It was determined to extract the fragments: two breaths of chloroform produced a mild anæsthesia, and fragments quite loose were removed, to a depth equal to the whole length of the fore-finger, and in the direction of the lower part of the right hemisphere. It was then felt necessary to stop; death took place in four days; and a large mass of the hemisphere was shown to be crushed and still interspersed thickly with fragments of the vitreous table. Dr. S.'s reflections led him to think that it was madness to admit the air to such a mass.

Another soldier received a wound which he himself described as insignificant. Two days after, the surgeon described it as "an unimportant wound by grazing." It was not minutely examined, but carefully bandaged. At one visit, the bandage was not removed, on account of the insignificance of the injury. In the third night, headache and stupor appeared, an examination was made, and a ball was found wedged in the parietal bone. The late Professor Langenbeck removed the ball with an elevator, and took away the sharp edges of the surrounding bone with a saw, and the loose pieces, partially driven into the brain, with a forceps. Not the slightest amelioration ensued, and in twenty-four hours (four days from the reception of the wound) death took place.

*The ball is split by the sharp, broken edges of the bone.*—A soldier received a shot in his left parietal bone. Three days after, while in a state of stupor, he was examined by Surgeon-General Niese, who reports the case. He immediately discovered half of the ball within the crown of the skull, an inch from the small wound of entrance. This was widened in the integuments, and a hole in the cranium discovered by doing so. The remaining half of the ball was close behind this, under the sharp edge of the opening in the skull, wedged so firmly that Dr. Niese could not remove it by direct efforts. He set the crown of a trepan directly over it, and removed it after perforating the cranium. (The removal of the first half is not particularized.)

No relief to the symptoms, even momentary, was apparent as the result of these operations. Under an antiphlogistic treatment, consciousness was, at intervals, restored. The patient died at the end of seven days. The dura mater and the brain were torn by splinters; and the crushed portions of the latter, in a large extent, softened and made to contain abscesses.

*The ball fractures both tables of the cranium without penetrating.*—These injuries are frequent; and, if they receive proper care and attention, generally terminate in recovery. They are seldom found except on the parietal bone, which affords more frequent occasion for the ball to glance, whether the face of the man receiving the wound be turned toward him who fires the gun, or in another direction. Shot wounds on the forehead, temples, or occiput are generally mortal on the field of battle; and where this is not the case, it can mostly be shown, from the appearance of the wound, that the impaction of the ball was oblique.

Principal Stromeyer has never seen cases in which both tables of the skull were simply fissured by a ball. When a ball strikes with force, it always indents the outer table; but he will not say that the fissure of both tables is theoretically impossible, if the cranium be brittle. The proper division of these injuries, therefore, is that which is derived from the amount of injury done in the inner table; whether fissures, slight depressions, or depression sufficient sensibly to encroach upon the cavity of the cranium.

*Fissure of the tabula vitrea.*—Dr. S. here compares these to the fissures revealed by indiscreet incisions, made after a blow, or a fall on the head. A ball, being of small weight and great velocity, shatters and indents the outer table, and then of necessity tends to indent the inner. Its pressure is so instantaneous that it cannot reach a wide surface, but is confined to the vicinity of the point of contact. A small, unyielding substance, driven by a hammer through a hard board, tends to cause the latter to burst open with some elasticity; a bullet knocks a piece out.

*Moderate indentation of both tables, with splintering of the inner.*—This occurs much more frequently than might be inferred from the considerations to which we have been thus far led. It is discovered by the trepan; or inferred from the symptoms being more severe than in the natural course of things, and indicating some additional cause.

*The depression is such as to show undeniably that the cavity of the skull suffers sensible constriction from it.*—It is almost without exception the case, that the depression is oblique, and deeper on one side; a circumstance which Dr. S. ascribes to the obliquity of the incidence of the ball. The lesions of the brain and dura mater can only be conjectured from the depth of the cavity in the bone, and the unfavorable course of the symptoms. There is no certain inference to be drawn relative to perforation, except from the presence of brain in the wound. From a wound of this description, inflicted by a grape-shot, he once found brain upon the sufferer's shoulder strap. The general symptoms on this point furnish no resource at all. Puncture of the dura mater does not depend upon the depth of the depression, but upon the projection downward of sharp-pointed fragments.

Dr. S. here apologizes for grouping together such very dissimilar changes in vital parts, an arrangement which he fears will not be considered as methodical; an apprehension certainly not needed. He is not a lecturer on pathology, but a master of military surgery, teaching us what actually occurs in some of the most important of all possible experience. The study of methodical classification should come first at the university; it is enough for the man of great practical authority to fit the already well-informed student for the duties assigned to him in his peculiar situation and responsibilities.

The *diagnosis* is easier than in civil practice, as there is always a wound present, which gives more or less opportunity of inspecting the wounded hard parts. A scrupulous examination of this, by sight and feeling, will rarely fail of betraying the internal injury. However slight the case appear, this should always be made; and done at the first opportunity. The actual danger is most formidable; and a minute examination keeps awake the attention of the surgeon. If delay be incurred, the handling of the part, or the application of instruments to it, is liable to inflict incalculable injury; and the chance of really useful information, from doing so, is entirely problematical.

Patients thus wounded were generally, but not always, prostrated to the ground. They soon, however, recovered from their stupefaction, could walk and get into a wagon, acquired some pain in the head and some giddiness on the road, and were generally relieved from these last symptoms when placed at rest, and subjected to cold applications. There were only a few who had vomiting on awakening from their stupefaction; and this was of short continuance. Those who had deep penetrating fragments, had more or less fixed pain in the injured part, and were more apt to be overcome with giddiness and a slight stupor than the others. Whatever accelerated the circulation, muscular efforts, or agitation, either emotional or of the senses, increased this tendency to the appearance of preoccupation of the mind and to pain in the head. In slighter cases the cephalic phenomena were often absent, until unfavorable agencies brought on congestion or inflammation. As a rule, no paralysis occurred; and the patient could move all his limbs in general, even when congestive or inflammatory phenomena had appeared. In individual cases, palsy appeared at the commencement; always on the opposite side. A pair of fingers, a hand, an arm, a pair of toes, a foot, a whole inferior extremity, or an upper and lower limb at the same time, were more or less deprived of power. These occurrences could be reduced to no exact anatomical or physiological connection with any particular locality. Of two injuries, in precisely the same part of the head, one was followed by a tedious incomplete paralysis of a thumb and index finger; and the other underwent the same affection in a great toe. "These observations," Dr. Stromeyer remarks, "the physiologist, if not himself a practitioner, must be content to receive from those who are such."

The *course* of these cases is exceedingly various, according to the attendant circumstances. Under favorable conditions, that is, with an antiphlogistic treatment, quiet and pure air, the symptoms are so slight as to give rise to the highest degree of astonishment. Dr. Stromeyer, in one place, speaks strongly of the disadvantage of the fear of blood-letting prevailing among the young men of the period, in Germany. This resistance to a doctrinal fashion which he finds erroneous in fact, is not to be misconstrued, as has been done in America, into the recommendation of extravagant abstractions of blood. He in no place lends the slightest countenance to any such practice; but his treatments are characterized by reason, moderation, and good sense, as much as by the profoundest skill. The imputation will not adhere to his character, whether it be used to promote what is called homœopathia, or the teachings of new doctrinal men within the legitimate profession.

Under this rational, unprejudiced, and disinterested treatment, no important congestive or inflammatory appearances occur, the wound does not swell much, is not very painful, and undergoes a healthy suppuration. In this are gradually loosened the deeper splinters of the outer table, the largest of which can generally be removed after the first week. Much later appear the fragments of the inner table. This repulsion of the splinters is partial throughout. In the outer table it generally embraces a wider surface than in the inner. In the latter it is confined to the loosest among those more deeply depressed. The fragments of this part, which are more closely connected with the rest of the cranium and with the dura mater, become rounded off at their points, and amalgamated together by a thin stratum of new bone. A small tumor, composed of these united materials, is formed in the cavity of the wound, leaving, however, at the deepest point, a vacancy communicating with the external air. This orifice seldom forms part of a direct canal. The contained mass forms a kind of shield, incomplete at one of its edges and somewhat larger than the external opening in the skull. The probe almost never reaches the dura mater by a direct route, but does so in a lateral direction. Whether this vacancy is ever filled with bone, appears to depend upon its size. The expectant treatment has the very important advantage that the brain, by that method, generally retains its protective covering of bone.

If the dura mater be pierced, the consequences, under a favorable line of conduct, are not so serious as would, *a priori*, be imagined. An adhesion very soon forms between the visceral and parietal folds of the arachnoid, by which the cavity of that membrane is closed; and the wounded dura mater becomes attached to the brain. The slow separation of the deeper splinters of the vitreous table allows time for the consolidation of these adhesions; one of the advantages of leaving them to the powers of nature. The healing process is more rapid than in ordinary blows and falls, from the circumstance that the adjacent parts, within a very limited distance, are uninjured.

*The ball fractures the inner table without injuring the outer.*—Our author has seen this injury but once. In one instance, besides, a scarcely perceptible fissure existed upon the external surface; and he satisfied several practiced ears of a difference of tone in tapping on the part in question and on adjacent spots. He diagnosticated a fracture of the inner table. The patient died of pyæmia, and the diagnosis was verified by dissection. The tone was higher in the musical scale, over such an internal splintering, than in other localities. He tapped with a silver probe.

He finds that this means of discrimination was known to Lanfranchi and Ambrose Paré.

Fragments may be driven in, to a small depth. This singular injury can be compared, as Dr. S. remarks, to the partial fracture of bones produced by pressure. The outer table has evidently more elasticity than the inner. This elasticity can be easily exemplified, as our author quotes from Hyrtl, by throwing a recent skull upon the floor, when it will rebound.

Many more of these would, no doubt, be discovered if the practice of trepanning to search for them were continued. There is no great presumption in assuming that their course, as a rule, would be favorable if the patient were kept for a sufficient length of time on an antiphlogistic diet; as these must evidently fare better than the preceding class, in which air had access to the fractured inner table. The older physicians labored under a great anxiety lest the fluids of the wound should not find an easy escape. They knew little of the comparative recovery of subcutaneous wounds, and those exposed to the atmosphere. They knew not how to explain what became of the secretions of the part. We now know that by protection from the air, and by a proper management, the fluids effused are reduced to a minimum, and no more of them is produced than suffices for the healing process; never, in the general rule, being so copious as to require an outlet. Within the "decade," as Principal Stromeyer remarks, this last has been thought necessary. Yet, he adds, the faithful and honorable details of cases by Percival Pott exhibit, in the clearest manner, that the admission of air to the wound is always followed by a great increase of suppuration. In most cases the increased secretion rendered a second trepanning necessary, and even a third; one operation thus becoming the cause of another.

The strongest indication for trepanning was thought to be stupor or insensibility; and it certainly, as is acknowledged by our distinguished author, requires a very solid conviction of duty in the case to refrain from it under such circumstances. It is so common to see the clearest consciousness instantly return on the elevation of a fragment, or the removal of an accumulation of blood. Yet we should recollect, he is of opinion, that typhus-fever patients have often lain for days and weeks in the deepest stupor, and yet gradually recovered the entire control of their mental faculties; and that trepanning, with the most entire success of its physical purpose, the elevation of a fragment or the removal of an effusion, in "interminably numerous cases," has exhibited absolutely no influence upon the return of sensibility; but this gradually took place under an antiphlogistic management. It requires that we should have actually viewed continuously the successful cure of wounds of the head without trepanning, to acquire the same secure and confident feeling of the anticipated results of our course that almost every physician feels with his typhus-fever cases.

We fear the following sentence will meet with opposition in the United States:—

"Would we not hold him a miserable quack who should still, for a mere soporose condition alone, want to give a typhus-fever patient musk, camphor, or assafœtida?"

It will not be long, he predicts, before men will pronounce no favorable opinion upon the surgeon who wishes to trepan for a soporose condition. For this he appeals with confidence to the numerous young physicians who witnessed the results of the Schleswig-Holstein campaigns of 1849 and 1850.

*The ball only injures the outer table.*—A number of cases occurred in which the ball carried away more or fewer pieces of the outer table and left a little lead in the diploe, without doing any further mischief. In a little time the fragment of the ball becomes loose, and can be removed.

In one case, a physician, who operated with levity, removed such a fragment with the trepan, and believed that he had left uninjured the vitreous table. Principal Stromeyer showed him the pulsations of the brain distinctly, at the bottom of the cavity he had made. Happily, the case terminated well.

*The ball simply bruises the skull.*—It is so notorious that contusions of the skull received in time of peace are often productive of dangerous subsequent effects, that one cannot help anticipating, *primâ facie*, that contusions from shot would have similar results. This, however, appears to be by no means the case. Dr. Stromeyer has seen only a single instance in which mere contusion of the skull from a ball was followed by death. Were we speaking of a less elevated and careful authority than our author, we should have said that this case, which he relates, is not an example of such an injury; the shot "cutting through" (durchschneidend) the sagittal suture. The case is very valuable; it is want of space that compels us to omit it.

Death from this cause is much less frequent in civil life in Germany than it formerly was, owing to systematic precaution in the medical men, and in a very great measure from the use of cold applications.

*The ball injures the integuments only.*—These hurts are generally contusions from flattened balls, grazing wounds with the loss of a piece of the scalp, or occasional "seton-wounds," in which the ball slips, for a short distance, under the scalp. The latter wounds are commonly, but not always, accompanied with injury to the bones. Sometimes a ball is arrested under the scalp.

In general, the discrimination of the injury done is rendered easier by the existence of an open wound; but these sometimes are not present, or are inflamed; in which case they cannot be completely examined without injury.

"It is not long," says Dr. Stromeyer, "since every surgeon thought it his holiest duty to make a crucial incision in every wound of the head, no matter how trifling was the injury." He urges, at some length, the impropriety and cruelty of such a practice; and concludes by alleging that it was unnecessary in his own large experience, and by almost leaning toward tolerating it as a preventive or cure of phlegmonous erysipelas of the scalp.

It sounds strange in the ear of a pupil of the Pennsylvania Hospital, and of the late Dr. Physick, to hear such a discussion, or an admission that this had been recently practiced. We had imagined, between 1814 and 1819, that such a mode of torture had been once and forever abandoned among civilized physicians; and we had not then heard of it as a remedy or preventive of erysipelas. The incisions of limited extent which were recommended and said to be still used by some practitioners, we were much inclined to consider of doubtful expediency, and fit never to be used except in very extreme and evident cases indeed, and never in any case that we saw. We supposed that in more crowded cities and hospitals, in besieged places, and during some epidemics of which we never witnessed examples, a different state of things might render this less degree of severity proper, though never so with us. It is something to have to boast, over learned, accomplished, and thoroughly-trained Germany, a little gratification of vanity such as this.

For the mere purpose of diagnosis, Dr. Stromeyer entirely reprobates this practice, and compares it to severe handling and giving unnecessary pain to a recent fracture, and to scraping off the pericranium from the skull and pouring ink on the bone to discover a fine fissure; an affectation of exactness, the result of which was to be of no value when obtained. An incomplete diagnosis in such a case is vastly better than the positive infliction of injury on the patient. For immemorial ages, men have known the existence of real danger in common blows on the head; yet the remedy for these, with watchfulness, is a dose of Epsom salts and the application of wet towels. In his own experience he has found cruelties unnecessary; and in all the Holstein wars has seen but one death from injury of the head where no portion of the cranium was shattered—the one which we have recorded above. The ball, he resumes, never produces so much concussion of the head as is customary in the falls and blows of civic life.

*Proportion of cases.*—At the battle of Idstedt, among 1200 wounded, 60 had shot wounds of the head, of which 31 were of the soft parts only; wounds of the head being thus in the ratio of five per cent. At the storm of Frederickstadt, of 500 wounded, 40 were hurt in the head, or eight per cent. In this case, the lower extremities of the men were very much protected by a dike.

A desire to be useful, in the present unhappy position of our country, induces the present writer to devote his remaining space almost exclusively to the treatment. First, however, he would wish to present the heads of the subjects treated of in the intervening space. Nothing is neglected by our conscientious author; nothing is forgotten. The subjects are,—Concussion, Compression, Congestive and Inflammatory Reaction, Traumatic Erysipelas of the Head, Traumatic Phlegmon of the Head, Traumatic Osteitis of the Cranium, Pyæmia, Phlebitis of the Encephalon, Secondary Encephalitis and Osteitis, Typhus with wounds of the Head, and Delirium Tremens with wounds of the Head.

*Comments on the treatment.*—It seems to have been the intention of our experienced author, in the preparation of his book, to leave himself at entire liberty to judge of the extent to which he is to carry any of the important inquiries to which he devotes it. He does not give us any entire system, any detailed formula of action, or any minute directions; and does not write unless he sees a benefit likely to be derived from the facts within his knowledge, or from his own reflections on them. We are supposed to have gained our systematic knowledge elsewhere; and his communications consist exclusively of criticisms. He occupies very considerable space in an examination of the opinions of celebrated practitioners on the expediency and indication of trepanning.*

As much as three hundred years ago, Lanfranchi gave as restricted rules for resorting to the trepan "as are now by Dieffenbach." Others, however, urged its application to the most extravagant length.

In 1811, Dr. S.'s father showed him a man with a large and deep indentation in his head, from a blow, healed without trepanning. Some time after, he saw Wedemeyer trepan a man with a depression as large as that made by a bullet, but which was the effect of a blow delivered by a piece of building stone, containing a projecting pebble. The man had no bad symptoms for three days; on the fourth he was trepanned, and he died on the fifth.

Dr. Stromeyer studied seven years, and attended hospitals for three years of the time, in Berlin, Paris, London, and Vienna; and never saw

---

* We refer some English critics to the following:—

We have seen no authority for *trephine* but Wiseman, who gives both words in the same sentence, and applies trephine to a differently-made instrument. *Trepan* has a regular Greek origin; τρύπανον and τρυπάνη, from τρυπάω, I bore. Τρύπανον is as old as Homer, Od., ix. 385, and is used by Hippocrates; certainly sufficient authority. Liddell and Scott quote it from Hipp. apud Galen. The curious may find it in Foes, ed. 1657, p. 544–54, de intern. affect., and 911 B, de cap. Vuln. *Trapani*, in Sicily, was undoubtedly within the Greek civilization.

a case recover by trepanning, during all that course of attendance! (Habe ich nie einen Kopfverletzen durch Trepanation heilen sehen.) Various severe instances recovered without the operation. In his first half year as a professor, he saved a laboring man, whom he trepanned for a complicated fracture. Near the same time, a little girl had her head pinched between two large stones. One side of the head was raised higher than the other; and a cavity produced in the surface, which held a tablespoonful of water. No operation was performed, and she got quite well, but the obliquity of the head was not removed. Dr. S. laid his trepanned cases before a scientific meeting at Erlangen, in 1840; on which occasion his friend Textor told him that, in his (M. Textor's) opinion, trepanning had become unnecessary; but as the latter did not show him how nature removed deep depressions, Dr. Stromeyer could not help receiving the dictum more as a paradox. Still, he never forgot it; and feels bound to do justice to his friend as to the matter in the present volume.

Since that time, he has only once performed the operation. This was on a boy twelve years old, in Munich. Part of both tables of the parietal bone had been crushed by a kick from a horse. Dr. Stromeyer removed the depressed portions of the outer table with a trepan of large size; and the inner, with a smaller one. The boy died; but Dr. S. believes that his death is to be ascribed to another kick, which produced a copious effusion of blood in the upper mediastinum, and impeded or paralyzed the action of the lungs.

In his service in the south of Germany, he had many cases of injury of the head; fewer in Kiel, in Holstein; which he ascribes to the gentle, careful, and self-controlled habits of the people.

From Messrs. Dease and Astley Cooper, and from statistics prepared by the honorable and judicious Brodie, he had become settled in the belief that complicated fractures of the cranium should be trepanned, to prevent the accumulation of pus. Yet he afterward found fractures of the skull heal so well that he began to think that, if it was in the power of treatment to prevent diffused suppuration in all cases, the aphorism of Brodie was either very elastic, or had lost all hold. It seemed to him demonstrated, that the access of air had a decidedly injurious influence.

Two more operations ensued in the hands of Professor Langenbeck, with a speedily fatal result. After the battle of Kolding, eight patients were brought to him with fractures of the cranium, depressions, and cerebral symptoms. In seven, the removal of the fragments was left to nature; and they all entirely recovered. The eighth continued quite insensible, and without diminution of his symptoms, for seven days. Dr. Stromeyer was then induced to dilate the wound and extract all the splinters. The dura mater was found to have received a small slit.

The suppression of the cerebral functions now became more striking; and an erysipelas of the face took place. The sequestrum was not removed until it was entirely loose. In five weeks, the stupor gradually left him; and he became quite well, gratifying his comrades by a flow of cheerful humor.

This treatment Dr. Stromeyer would call neither operative nor expectant. It was *antiphlogistic*. Some of our readers may be reminded that this does not imply abstraction of blood. Dr. S. recommended it to the young surgeons under his direction; and was happy to find that all the wounds by small arms, in which injury of the dura mater and brain was doubtful, or was only inferred from the depth of the depression, although the patients might have lain in stupor for weeks, got well, without either palsy or impairment of the faculties.

The preceding is all that our space and time will permit us to introduce of the experience and judgment of this great practitioner. The disastrous crisis which we deprecated in the early part of the first article of this review, has now come upon the American Union; and the present writer finds it difficult to believe that any lover of his country, or any true physician, can other than wish the publication, as soon as practicable, of a good translation of this invaluable work. He begs leave to announce that he has the task of converting it into our language now in hand.

